# Impaired glucose transporter-1 degradation and increased glucose transport and oxidative stress in response to high glucose in chondrocytes from osteoarthritic versus normal human cartilage

**DOI:** 10.1186/ar2713

**Published:** 2009-06-02

**Authors:** Susana C Rosa, Juliana Gonçalves, Fernando Judas, Ali Mobasheri, Celeste Lopes, Alexandrina F Mendes

**Affiliations:** 1 Center for Neurosciences and Cell Biology, and Faculty of Pharmacy, University of Coimbra, 3004-517 Coimbra, Portugal; 2 Orthopaedics Department, University Hospital of Coimbra, Avenida Bissaya Barreto, Bloco de Celas, 3000-075 Coimbra, Portugal; 3 Division of Veterinary Medicine, School of Veterinary Science and Medicine, Sutton Bonington Campus, University of Nottingham, Sutton Bonington LE12 5RD, UK

## Abstract

**Introduction:**

Disorders that affect glucose metabolism, namely diabetes mellitus (DM), may favor the development and/or progression of osteoarthritis (OA). Thus far, little is known regarding the ability of chondrocytes to adjust to variations in the extracellular glucose concentration, resulting from hypoglycemia and hyperglycemia episodes, and so, to avoid deleterious effects resulting from deprivation or intracellular accumulation of glucose. The aim of this study was to compare the ability of normal and OA chondrocytes to regulate their glucose transport capacity in conditions of insufficient or excessive extracellular glucose and to identify the mechanisms involved and eventual deleterious consequences, namely the production of reactive oxygen species (ROS).

**Methods:**

Chondrocytes, isolated from normal and OA human cartilage, were maintained in high-density monolayer cultures, in media without or with 10 or 30 mM glucose. Glucose transport was measured as the uptake of 2-deoxy-D-glucose (2-DG). Glucose transporter-1 (GLUT-1) mRNA and protein content were evaluated by real-time RT-PCR and western blot, respectively. ROS production was measured with 2',7'-dichlorodihydrofluorescein diacetate.

**Results:**

Basal and IL-1β-induced 2-DG uptake, including the affinity (1.066 ± 0.284 and 1.49 ± 0.59 mM) and maximal velocity (0.27 ± 0.08 and 0.33 ± 0.08 nmol/μg protein/hour), and GLUT-1 content were identical in normal and OA chondrocytes. Glucose deprivation increased 2-DG uptake and GLUT-1 protein both in normal and OA chondrocytes. Exposure to high glucose (30 mM) for 18 or 48 hours decreased those parameters in normal but not in OA chondrocytes. GLUT-1 mRNA levels were unaffected by high glucose, either in normal or OA chondrocytes. The high glucose-induced reduction in GLUT-1 protein in normal chondrocytes was reversed by treatment with a lysosome inhibitor. High glucose induced ROS production, which lasted significantly longer in OA than in normal chondrocytes.

**Conclusions:**

Normal human chondrocytes adjust to variations in the extracellular glucose concentration by modulating GLUT-1 synthesis and degradation which involves the lysosome pathway. Although capable of adjusting to glucose deprivation, OA chondrocytes exposed to high glucose were unable downregulate GLUT-1, accumulating more glucose and producing more ROS. Impaired GLUT-1 downregulation may constitute an important pathogenic mechanism by which conditions characterized by hyperglycemia, like DM, can promote degenerative changes in chondrocytes that can facilitate the progression of OA.

## Introduction

Osteoarthritis (OA) is the most common musculoskeletal disorder and a major cause of disability that affects diarthrodial joints, being characterized by cartilage degradation, accompanied by local inflammation and changes in the subchondral bone. Increasing age, excessive loading or injury, genetic predisposition and obesity are important risk factors for the development and progression of OA [1,2]. Present evidence, including epidemiologic studies, suggests the existence of a positive correlation between OA and conditions that affect glucose metabolism; namely, glucose imbalance, metabolic dysfunction and diabetes mellitus (DM) [3-6]. The association between DM and OA has already been suggested in early epidemiologic studies that showed a higher incidence of radiographic OA, with an earlier onset and more severe manifestations, in diabetic patients [7]. Nevertheless, the fact that the incidence of both OA and DM, especially type 2 DM, increases with age raises the possibility that these two conditions coexist by chance alone [8]. In this case, whether the metabolic and systemic disturbances, due to hyperglycemia and/or altered insulin plasma levels characteristic of DM, have consequences in joint tissues is largely unknown, but several mechanisms may contribute to aggravate OA and promote its progression, especially in type 2 DM patients [9].

Despite improved therapeutic possibilities, strict control of glycemia in diabetic patients is still impossible, so that hyperglycemia and, less frequently, hypoglycemic episodes occur in those patients [10]. Since fully developed articular cartilage is an avascular tissue, glucose reaches chondrocytes through diffusion from the synovial fluid [11], where its concentration is identical to and reflects that in the plasma, both under normal conditions and in noninflammatory and inflammatory types of arthritis, excluding those associated with infections [12]. To our knowledge, no information is available comparing the synovial fluid and plasma glucose concentrations in diabetic patients, and, despite many possible complicating factors, DM-related variations in glycemia are likely to cause similar changes in the synovial fluid glucose concentration, and thus affect glucose delivery to the articular cartilage. Articular chondrocytes are highly glycolytic cells, requiring a steady supply of glucose for optimal energy production and cell homeostasis, as well as for anabolic functions; namely, the synthesis of cartilage matrix molecules [1]. As such, articular chondrocytes may be especially sensitive to alterations in the synovial fluid glucose concentration due to hypoglycemia and/or hyperglycemia episodes.

Studies evaluating the role of high and low extracellular glucose concentrations in articular chondrocyte functions are scarce, but glucose deprivation or inhibition of its uptake were shown to increase the expression of matrix metalloproteinase-2 [13], an enzyme that contributes to cartilage degradation in late OA. In another study, exposure to either low or high glucose concentrations induced insulin-like growth factor-1 resistance and decreased proteoglycan synthesis, which may constitute important pathogenic mechanisms in OA [14]. Exposure to elevated glucose concentrations was also shown to decrease dehydroascorbate transport into chondrocytes, which can compromise the synthesis of type II collagen [15]. Furthermore, in intervertebral disc cells, which share many common phenotypic characteristics with articular chondrocytes, glucose deprivation has been shown to reduce the synthesis of type II collagen [16], which is the major collagen in the articular cartilage matrix [1].

The molecular mechanisms involved in the effects reported in those studies were not elucidated, but increased production of reactive oxygen species (ROS) has been shown to mediate the damaging effects of hyperglycemia in various cell types [17]. Moreover, ROS contribute to the pathogenesis of OA by mediating many of the effects induced by catabolic cytokines, such as IL-1β, in articular chondrocytes [1]. Among other responses, ROS have been shown to decrease the synthesis and induce the degradation of cartilage matrix proteins [18], to promote cell death [19], and to alter the regulation of transcription factors such as activator protein-1 [20] and NF-κB [21] that are involved in cartilage degradation and joint inflammation [1,22].

Facilitated glucose transport represents the first rate-limiting step in glucose utilization by chondrocytes, and thus may contribute to any effects due to changes in plasma and synovial glucose concentrations. Several members of the facilitative glucose transporter family – the GLUT/SLC2A transporters – have been identified in human articular chondrocytes, among which glucose transporter-1 (GLUT-1) is especially important as it is regulated by both anabolic and catabolic stimuli, while others, like glucose transporter-3, are constitutively expressed and unaffected by those stimuli [13,23-25]. In addition, various cell types have been shown to adjust to high and low glucose concentrations, mimicking hyperglycemia and hypoglycemia episodes, by changing the GLUT-1 content and the rate of glucose transport [26-28]. Moreover, a recent study reported that glucose uptake in equine chondrocytes represents a constant fraction of the glucose concentration in the culture medium [29], implying that glucose transport in these cells depends on the extracellular glucose concentration. Whether and how human chondrocytes can also adjust their glucose transport capacity to changes in the extracellular glucose concentration, and whether modulation of GLUT-1 content is involved, remain to be elucidated.

The aim of the present study was therefore to determine and compare the ability of normal and OA chondrocytes to modulate the GLUT-1 content and glucose transport in response to high and low extracellular glucose concentrations, since failure to do so may cause cell damage and affect chondrocyte functions, contributing to the development and progression of OA, especially in DM patients. Since the results obtained showed that OA chondrocytes, unlike their normal counterparts, are unable to downregulate the GLUT-1 content and glucose transport when exposed to high glucose concentrations, the molecular mechanisms involved in GLUT-1 downregulation were investigated. Furthermore, and to determine whether altered glucose transport in OA chondrocytes under high glucose conditions can have deleterious consequences on their functions, the production of ROS was compared in normal and OA chondrocytes.

## Materials and methods

### Cartilage samples and chondrocyte culture

Human knee cartilage was collected from the distal femoral condyles of 15 multiorgan donors (28 to 35 years old, mean age 31 years; normal cartilage) or with informed consent from 18 patients (52 to 77 years old, mean age 66 years; OA cartilage) undergoing total knee replacement surgery at the Orthopedic Department of the University Hospital of Coimbra. The Ethics Committee of the University Hospital of Coimbra approved all of the procedures.

Chondrocytes were isolated by enzymatic digestion as described previously [30]. Nonproliferating monolayer cultures were established from each cartilage sample, allowed to recover in medium containing 5% fetal bovine serum for 24 hours, serum-starved overnight and maintained thereafter in serum-free culture medium. The cells were subsequently cultured, for the periods indicated in the figure legends, in glucose-free DMEM (glucose deprivation), Ham's F-12 (regular glucose medium (RGM), which contains 10 mM glucose) or Ham's F-12 supplemented with 20 mM D-glucose to yield a final glucose concentration of 30 mM (high glucose medium). In selected experiments described in the Results section, Ham's F-12 was supplemented with 20 mM mannitol to determine whether the observed responses to high glucose were due to osmotic effects. Recombinant human IL-1β 30 ng/ml (Peprotech, Rocky Hill, NJ, USA), the proteasome inhibitor, MG-132 10 μM (Calbiochem, La Jolla, CA, USA), and the lysosome inhibitor, chloroquine 20 μM (Sigma Chemical Co., St Louis, MO, USA), were added to the chondrocyte cultures as indicated in the Results section and the figure legends.

### 2-Deoxy-D-glucose uptake assay

Glucose transport was determined by measuring the net uptake of 2-deoxy-D-glucose (2-DG) (Sigma Chemical), a nonmetabolizable analogue of glucose. Briefly, chondrocytes were incubated in glucose-free DMEM containing 0.5 mM 2-DG and 0.5 μCi/ml [2,6-^3^H]-2-DG (GE Healthcare, Little Chalfont, UK) with a specific activity of 53 Ci/mmol, at 37°C for 30 minutes, in the presence or absence of cytochalasin B 10 μM (Calbiochem), a specific inhibitor of the majority of the facilitative glucose transporters, to determine the nonspecific uptake. The affinity and maximal velocity of 2-DG uptake were deduced from Michaelis–Menten plots obtained with 2-DG concentrations ranging from 0 to 5 mM. For each sample, the nonspecific uptake was subtracted from the total uptake, after normalization to the respective protein concentration.

### Western blot analysis

Whole cell lysates were prepared in RIPA buffer and the protein concentration was measured using the bicinchoninic acid/copper (II) sulphate protein assay kit (Sigma Chemical). The samples (25 μg protein) and molecular weight markers (All blue, Precision Plus molecular weight markers; Bio-Rad Laboratories Inc., Hercules, CA, USA) were subjected to SDS-PAGE and electroblotted onto polyvinylidene difluoride (PVDF) membranes, which were probed with a rabbit polyclonal antibody to human GLUT-1 (1:4,000 dilution; FabGennix Inc. International, Frisco, TX, USA) and then with an anti-rabbit alkaline phosphatase-conjugated secondary antibody (1:20,000 dilution; GE Healthcare). Immune complexes were detected with the Enhanced ChemiFluorescence reagent (GE Healthcare) in a Storm 840 scanner (GE Healthcare). A mouse anti-actin monoclonal antibody (1:10,000 dilution; Millipore Corporation, Billerica, MA, USA) was used to measure the expression of this housekeeping gene product as an internal control. The intensity of the bands was analyzed using ImageQuant™ TL (GE Healthcare).

### Total RNA extraction and quantitative real-time RT-PCR

Total RNA was extracted with TRIzol (Invitrogen, Paisley, UK), analyzed using Experion RNA StdSens Chip (Bio-Rad Laboratories) and quantified in a NanoDrop ND-1000 Spectrophotometer (NanoDrop Technologies, Inc., Wilmington, DE, USA) at 260 nm. The cDNA was reverse transcribed using the iScript™ Select cDNA Synthesis Kit (Bio-Rad Laboratories). Specific sets of primers for GLUT-1 and endogenous control genes were designed using Beacon Designer software (PREMIER Biosoft International, Palo Alto, CA, USA). Details of the forward and reverse primers for the genes evaluated are presented in Table 1. Quantitative real-time RT-PCR was performed with iTaq™ DNA polymerase using iQ™ SYBR Green Supermix (BioRad Laboratories).

**Table 1:** Oligonucleotide primer pairs used for quantitative real-time RT-PCR

Gene	Primer sequence (5'-3')	Product length (base pairs)	GenBank accession number
Glucose transporter-1	Forward: CGTCTTCATCATCTTCACTG	148	[Genbank:NM_006516]
	Reverse: CTCCTCGGGTGTCTTATC		
β-Actin	Forward: AACTACCTTCAACTCCAT	161	[Genbank:NM_001101]
	Reverse: TGATCTTGATCTTCATTGTG		
Cyclophilin A	Forward: CAGTCCCAGGAAGTGTCAATG	155	[Genbank:NM_021130]
	Reverse: CAGCGTCTCACTATGTTGCC		

The efficiency of the amplification reaction for each gene was calculated by running a standard curve of serially diluted cDNA sample, and the specificity of the amplification products was checked by analysis of the melting curve. Gene expression changes were analyzed using the built-in iQ5 Optical system software version 2, which enables the analysis of the results with the Pfaffl method, a variation of the ΔΔCT method corrected for gene-specific efficiencies. The results for GLUT-1 were normalized using two housekeeping genes, β-actin and cyclophilin A, determined with Genex^® ^software (MultiD Analyses AB, Göteborg, Sweden) as the most stable under the experimental conditions used.

### Measurement of reactive oxygen species production

The intracellular production of ROS was measured using 2',7'-dichlorodihydrofluorescein diacetate (Molecular Probes, Eugene, OR, USA) – a nonfluorescent probe that diffuses freely into cells, being hydrolyzed by intracellular esterases to 2',7'-dichlorodihydrofluorescein, which is cell membrane impermeable. In the presence of ROS, 2',7'-dichlorodihydrofluorescein is oxidized to 2',7'-dichlorofluorescein, a highly fluorescent compound.

After culture in RGM or high glucose medium for the periods indicated in the figure legends, chondrocytes were loaded with 5 μM 2',7'-dichlorodihydrofluorescein diacetate in PBS (pH 7.4) for 20 minutes at 37°C and resuspended in PBS. The fluorescence intensity was measured immediately using a fluorometer (LS50B; Perkin-Elmer, Waltham, MA, USA), with excitation set at 495 nm and emission set at 520 nm. The cell suspensions were then centrifuged and lysed in 10 mM Tris–HCl, 10 mM NaCl, 3 mM MgCl_2_, 0.5% Nonidet P-40, protease inhibitors (Roche, Indianapolis, IN, USA), pH 7.5. The protein concentration of the supernatants was measured using the bicinchoninic acid/copper (II) sulfate protein assay kit (Sigma Chemical). The fluorescence intensity of each sample was normalized to the total protein content.

### Statistical analysis

Statistical significance was assessed by two-way analysis of variance followed by a Bonferroni post test and an unpaired Student's *t *test for multiple and single comparisons, respectively, using GraphPad Prism version 5.00 (GraphPad Software, San Diego, CA, USA).

## Results

### Basal and IL-1β-induced glucose transport and GLUT-1 content in normal and osteoarthritis chondrocytes

**Figure 1:**
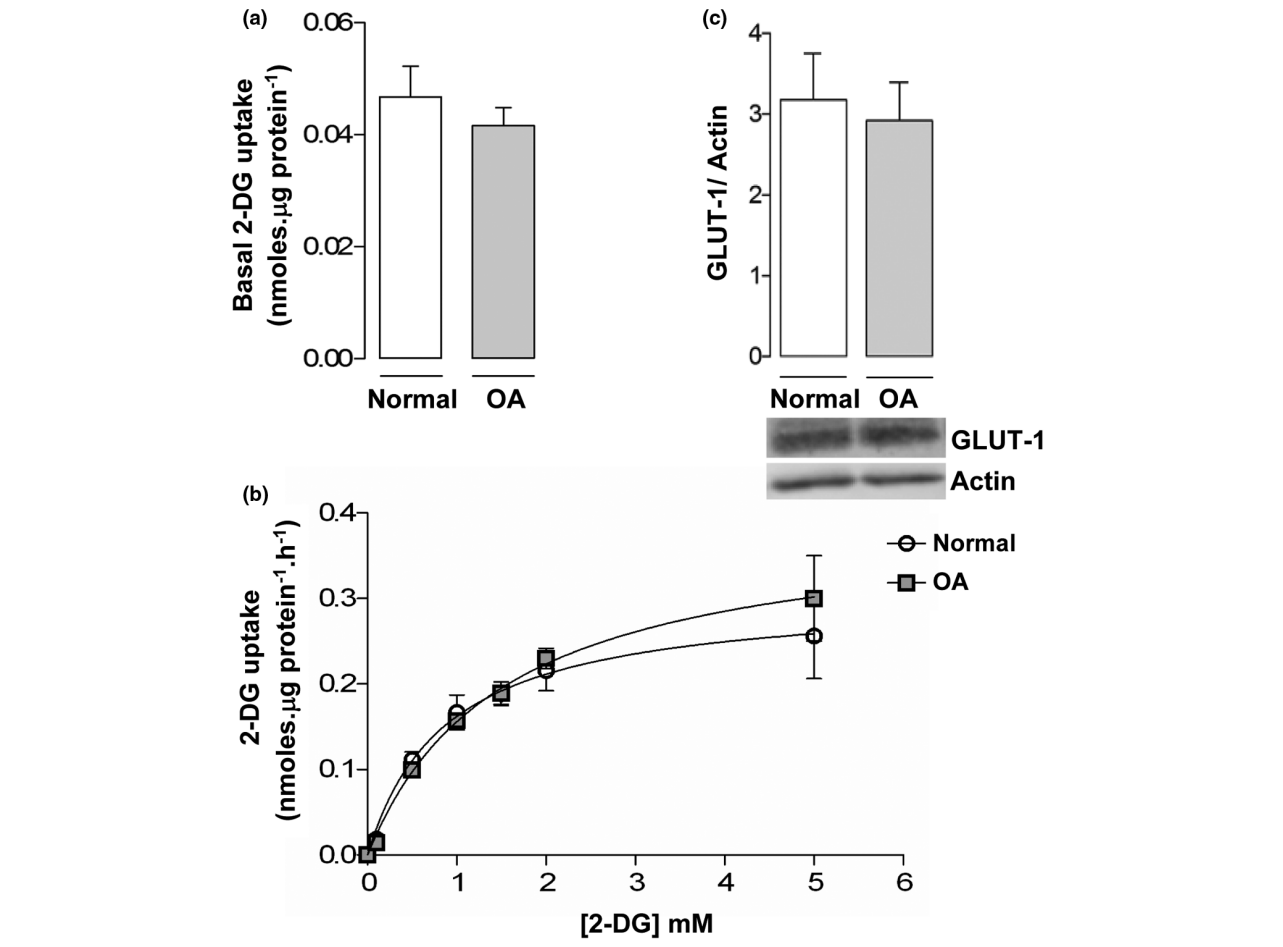
Basal glucose transport and glucose transporter-1 protein in normal and osteoarthritis chondrocytes. **(a) **2-Deoxy-D-glucose (2-DG) transport into normal (n = 6) and osteoarthritis (OA) (n = 9) chondrocytes. **(b) **Concentration dependence of 2-DG influx into normal and OA chondrocytes fitted to the Michaelis–Menten model to determine the affinity and maximal velocity. Each value is the mean ± standard deviation of five independent experiments performed in duplicate. **(c) **Glucose transporter-1 (GLUT-1) protein normalized to the respective actin band in normal (n = 9) and OA (n = 9) chondrocyte cultures. Bars = mean ± standard deviation.

Figure 1a reveals that the basal 2-DG uptake was identical in normal and OA chondrocytes. Furthermore, cytochalasin B inhibited 2-DG uptake by approximately 90% (data not shown), suggesting that glucose transport is almost entirely mediated by glucose transporters both in normal and OA chondrocytes. The intrinsic activities of the glucose transporters in normal and OA chondrocytes were also similar, as indicated by the analogous values for affinity (1.07 ± 0.28 and 1.49 ± 0.59 mM, respectively) and maximal velocity (0.27 ± 0.08 and 0.33 ± 0.08 nmol/μg protein/hour, respectively) obtained from the Michaelis–Menten plots presented in Figure 1b. Accordingly, GLUT-1 protein content did not differ significantly between the normal and OA chondrocyte cultures (Figure 1c).

**Figure 2:**
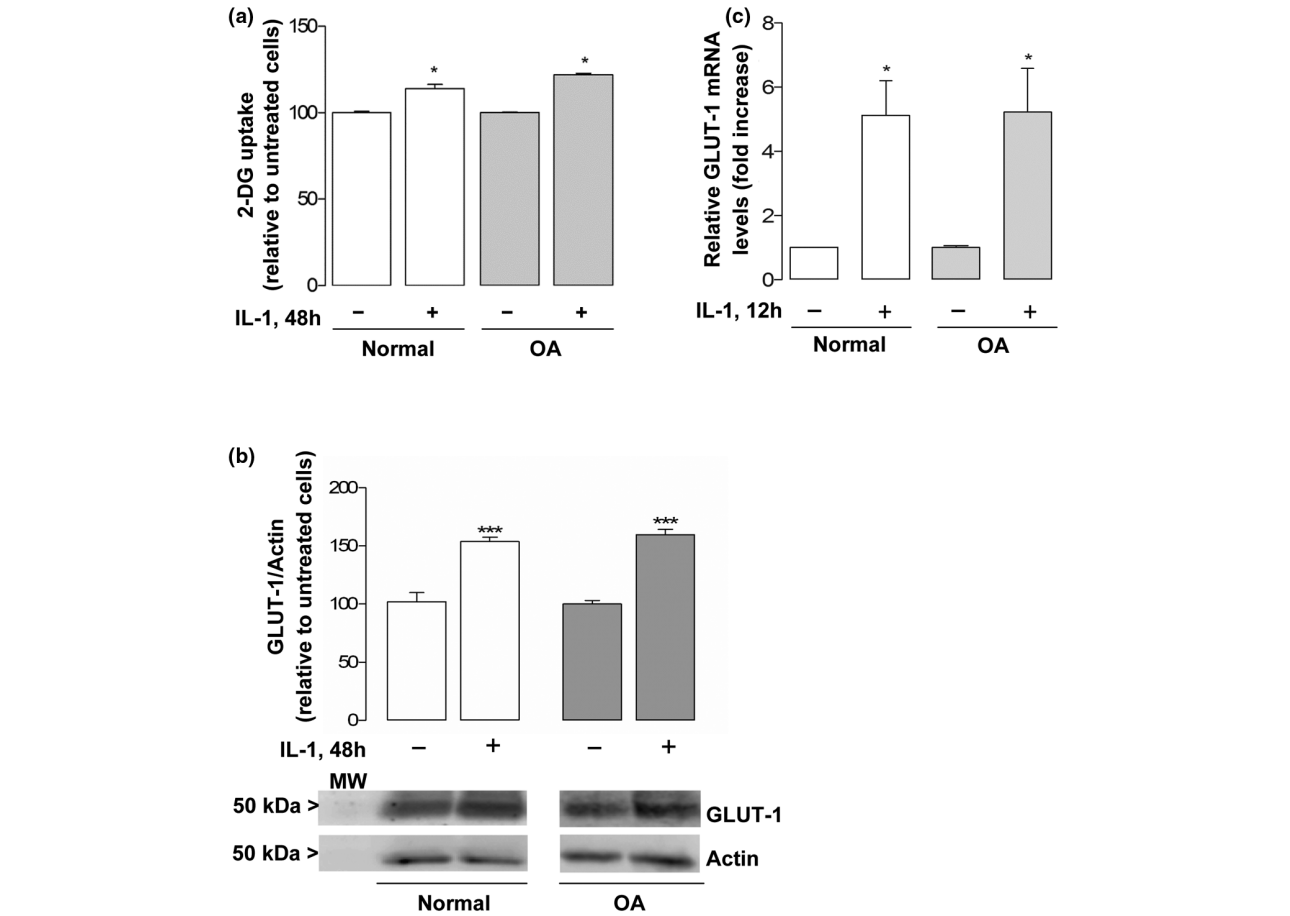
Stimulation of glucose transport and glucose transporter-1 expression by IL-1β in normal and osteoarthritis chondrocytes. **(a) **2-Deoxy-D-glucose (2-DG) transport into normal (n = 4) and osteoarthritis (OA) (n = 5) chondrocytes stimulated or not with IL-1β 30 ng/ml for 48 hours. **(b) **Glucose transporter-1 (GLUT-1) protein normalized to the respective actin band in normal (n = 4) and OA (n = 9) chondrocyte cultures, stimulated or not with IL-1β 30 ng/ml for 48 hours. Results expressed as the percentage relative to the respective control cells. MW, molecular weight marker. **(c) **GLUT-1 mRNA levels in normal (n = 3) and OA (n = 3) chondrocyte cultures stimulated or not with IL-1β 30 ng/ml for 12 hours. Results are expressed as the fold increase relative to the respective untreated cells. *P < 0.05 and ***P < 0.001 relative to untreated cells. Bars = mean ± standard deviation.

Upon stimulation with IL-1β 30 ng/ml, the 2-DG uptake and GLUT-1 protein and mRNA levels increased similarly in normal and OA chondrocytes, relative to the respective untreated cells (Figure 2). This indicates that OA chondrocytes regulate glucose transport and GLUT-1 levels in response to IL-1β as efficiently as their normal counterparts.

### Modulation of glucose transport by the extracellular glucose concentration in normal and osteoarthritis chondrocytes

**Figure 3:**
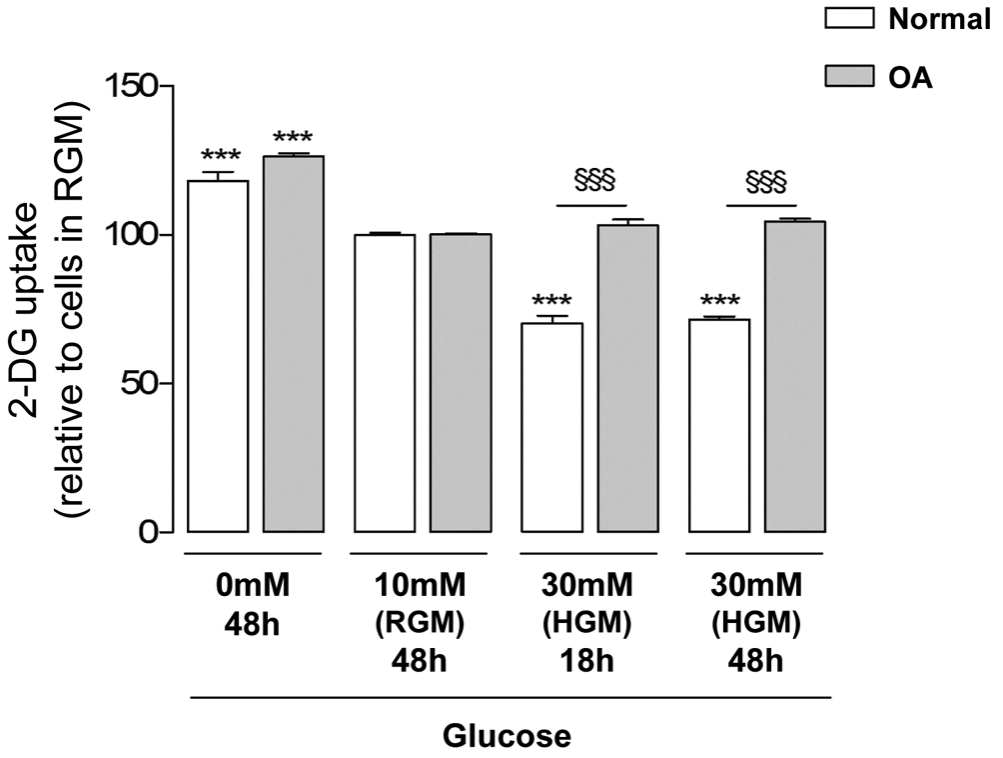
Modulation of glucose transport by different extracellular glucose concentrations. 2-Deoxy-D-glucose (2-DG) uptake into normal (n = 6) and osteoarthritis (OA) (n = 9) chondrocytes cultured in media with 0 mM, 10 mM (regular glucose medium (RGM)) or 30 mM glucose (high glucose medium (HGM)) for 18 or 48 hours. Results expressed as the percentage relative to the respective control cells maintained in RGM. ***P < 0.001 relative to cells maintained in RGM, ^§§§^*P *< 0.001 between normal and OA chondrocytes exposed to the same glucose concentration for the same period. Bars = mean ± standard deviation.

Normal and OA chondrocytes responded similarly to glucose deprivation, significantly increasing the 2-DG uptake relative to cells maintained in RGM (10 mM glucose) (Figure 3). In contrast, 2-DG uptake by normal chondrocytes cultured under high (30 mM) glucose concentrations for either 18 or 48 hours was approximately 30% lower than that found in their respective controls, that is, normal chondrocytes maintained in RGM for 48 hours. On the contrary, the 2-DG uptake in OA chondrocytes subjected to high glucose for either 18 or 48 hours did not change relative to their respective control cells cultured in RGM for 48 hours, but was significantly higher than that found in their normal counterparts cultured under high glucose concentrations for the same periods of time.

To control for possible osmotic effects, normal and OA chondrocytes were cultured in Ham's F-12 medium supplemented with 20 mM mannitol. In this condition, no changes in 2-DG uptake were found either in normal or OA chondrocytes relative to the respective control cells cultured in RGM (data not shown).

### Modulation of GLUT-1 protein content by the extracellular glucose concentration in normal and osteoarthritis chondrocytes

**Figure 4:**
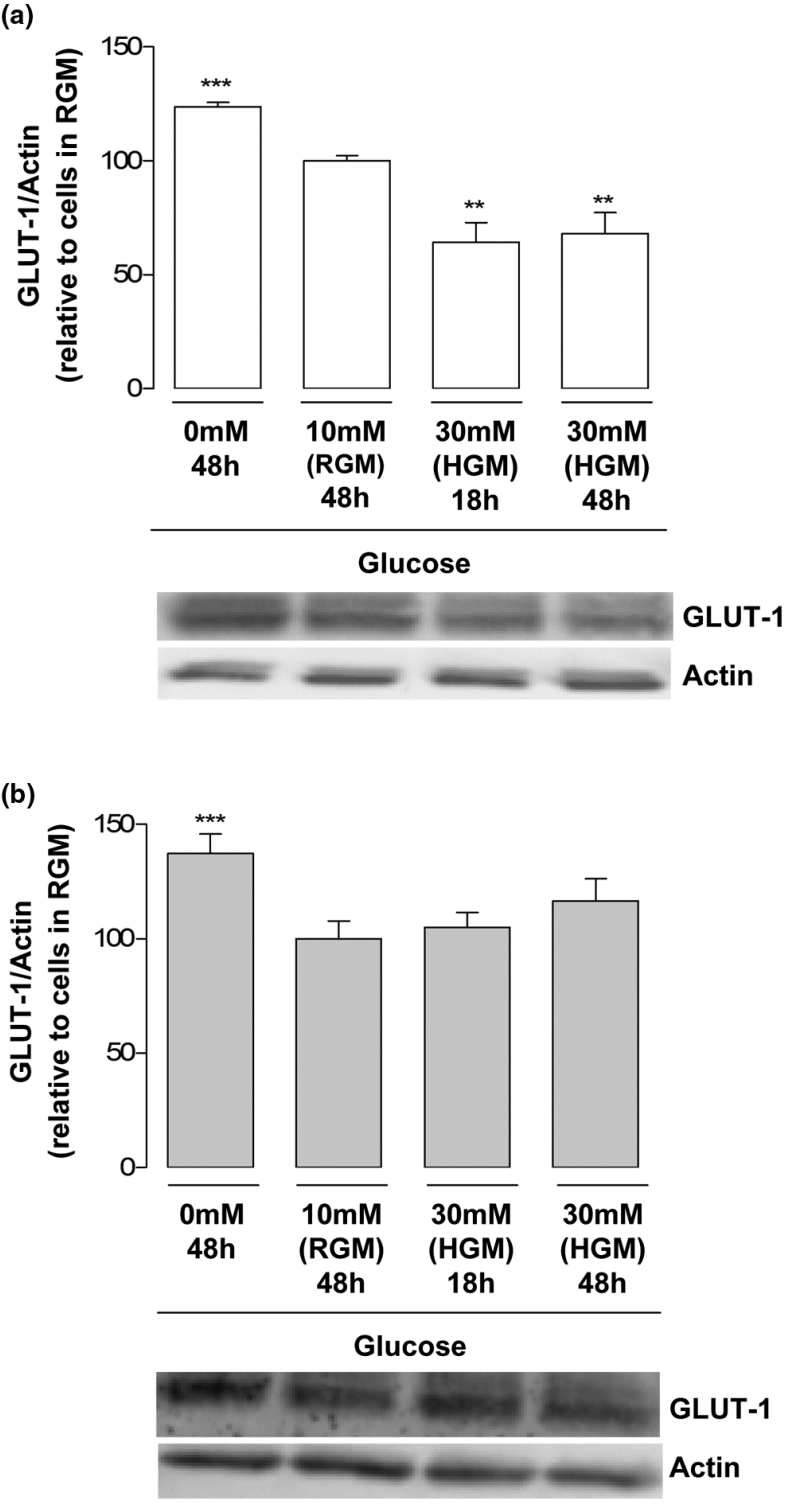
Modulation of glucose transporter-1 protein content by different extracellular glucose concentrations. Glucose transporter-1 (GLUT-1) protein normalized to the respective actin band in chondrocytes cultured in media with 0 mM, 10 mM (regular glucose medium (RGM)) or 30 mM glucose (high glucose medium (HGM)) for 18 or 48 hours. **(a) **Normal chondrocytes (n = 4). **(b) **Osteoarthritis chondrocytes (n = 6). Results expressed as the percentage relative to the respective control cells maintained in RGM. **P < 0.01 and ***P < 0.001 relative to cells maintained in RGM. Bars = mean ± standard deviation.

Glucose deprivation for 48 hours significantly increased the total GLUT-1 protein levels both in normal chondrocytes (Figure 4a) and in OA chondrocytes (Figure 4b), relative to their respective controls cultured under RGM for the same period. Since this increase (approximately 30%), either in normal or OA chondrocytes, is of the same magnitude as that found for glucose uptake (approximately 25%), it is likely to account for most, if not all, of the extra glucose transport capacity induced by glucose deprivation.

The total GLUT-1 protein content was markedly decreased in normal chondrocytes incubated with 30 mM glucose for 18 or 48 hours (Figure 4a), but remained unchanged in OA cells cultured under the same conditions (30 mM glucose), relative to those cultured in RGM, independently of the duration of exposure to high glucose (Figure 4b).

As regards 2-DG uptake, no differences were found in the GLUT-1 protein content in normal and OA chondrocytes cultured in mannitol-supplemented medium relative to their respective control cells maintained in RGM (data not shown).

### Role of high extracellular glucose on GLUT-1 mRNA levels

**Figure 5:**
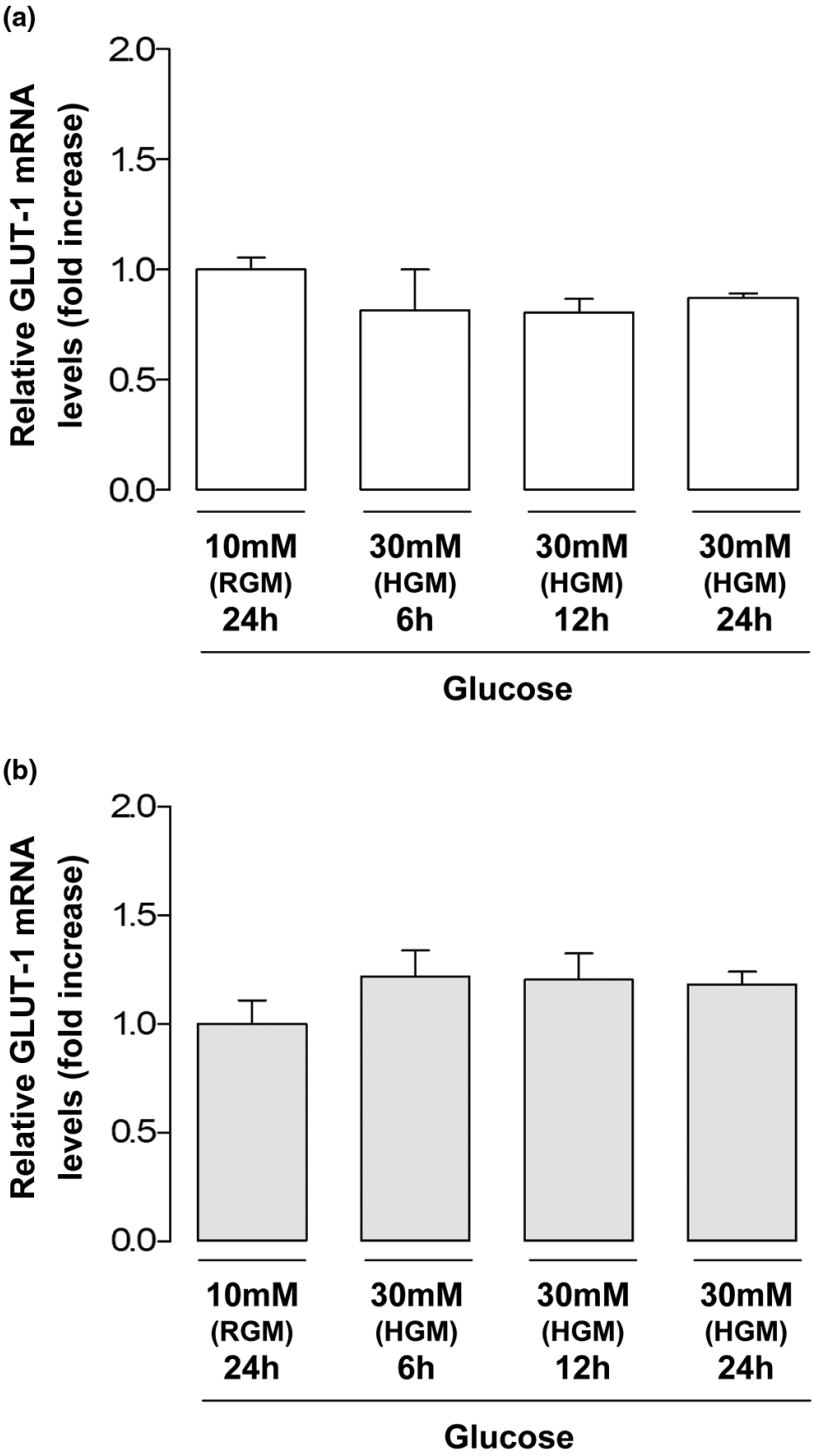
Regulation of glucose transporter-1 mRNA levels by high glucose. Quantitative real-time RT-PCR analysis of glucose transporter-1 (GLUT-1) mRNA levels in chondrocyte cultures exposed to 30 mM glucose (high glucose medium (HGM)) for 6, 12 or 24 hours or maintained in regular glucose medium (RGM). **(a) **Normal chondrocytes (n = 3). **(b) **Osteoarthritis chondrocytes (n = 3). Results expressed as the fold increase relative to the respective control cells maintained in RGM. Bars = mean ± standard deviation.

To ascertain whether the differences in total GLUT-1 protein content induced by culture of normal and OA chondrocytes under high glucose were due to alterations in GLUT-1 gene expression, quantitative real-time RT-PCR analysis was performed. The results obtained show that GLUT-1 mRNA levels, expressed as the fold increase relative to the respective control cells maintained in RGM, were not affected by culture of either normal or OA chondrocytes with high glucose for 6, 12 or 24 hours (Figure 5).

### Role of the lysosome and the proteasome on GLUT-1 downregulation

**Figure 6:**
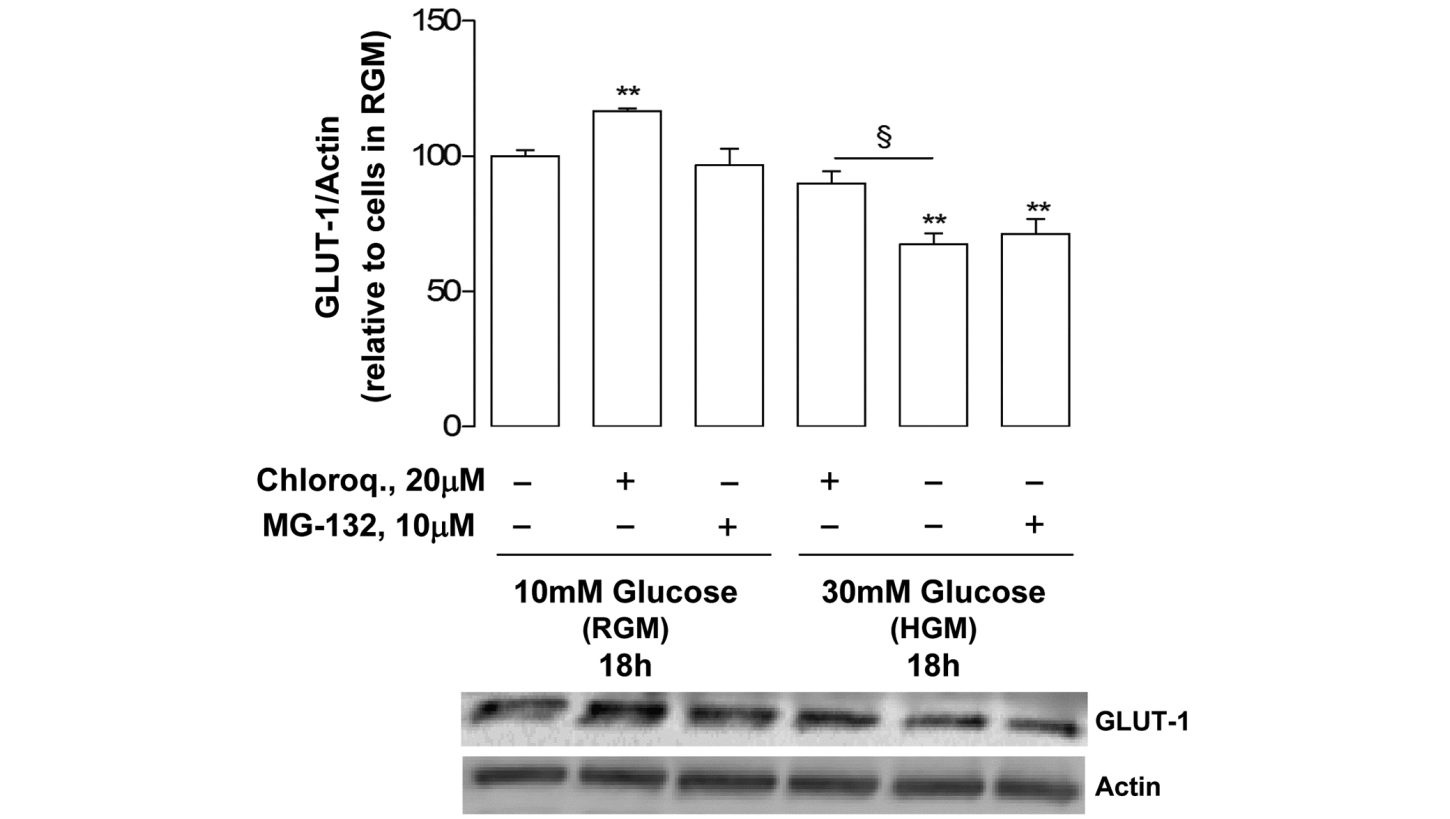
Roles of the proteasome and the lysosome in mediating high-glucose-induced downregulation of glucose transporter-1 protein. Glucose transporter-1 (GLUT-1) protein content normalized to the respective actin band in normal chondrocytes (n = 3) cultured in regular glucose medium (RGM) or in high glucose medium (HGM, 30 mM) with or without 20 μM chloroquine or 10 μM MG-132 added for the last 6 hours of a total 18-hour incubation period. Results expressed as the percentage relative to untreated cells maintained in RGM. **P < 0.01 relative to cells maintained in RGM, ^§^*P *< 0.01 between glucose 30 mM with or without 20 μM chloroquine. Bars = mean ± standard deviation.

To determine the contribution of the major protein degradation pathways to the decrease in the total GLUT-1 protein content found in normal chondrocytes exposed to high glucose (Figure 4a), specific inhibitors of the proteasome (MG-132) and lysosome (chloroquine) were used. Since both inhibitors were toxic to chondrocytes for periods longer than 6 hours (data not shown), they were added to the chondrocyte cultures only for the last 6 hours of a total 18-hour incubation period in the presence of 30 mM glucose. Treatment of normal chondrocytes cultured under high glucose with 20 μM MG-132 had no effect on GLUT-1 protein levels, whereas 20 μM chloroquine partially reversed the high-glucose-induced GLUT-1 decrease, augmenting GLUT-1 protein by approximately 20%, relative to chondrocytes cultured in the absence of this inhibitor (Figure 6).

### High-glucose-induced reactive oxygen species production in normal and osteoarthritis chondrocytes

**Figure 7:**
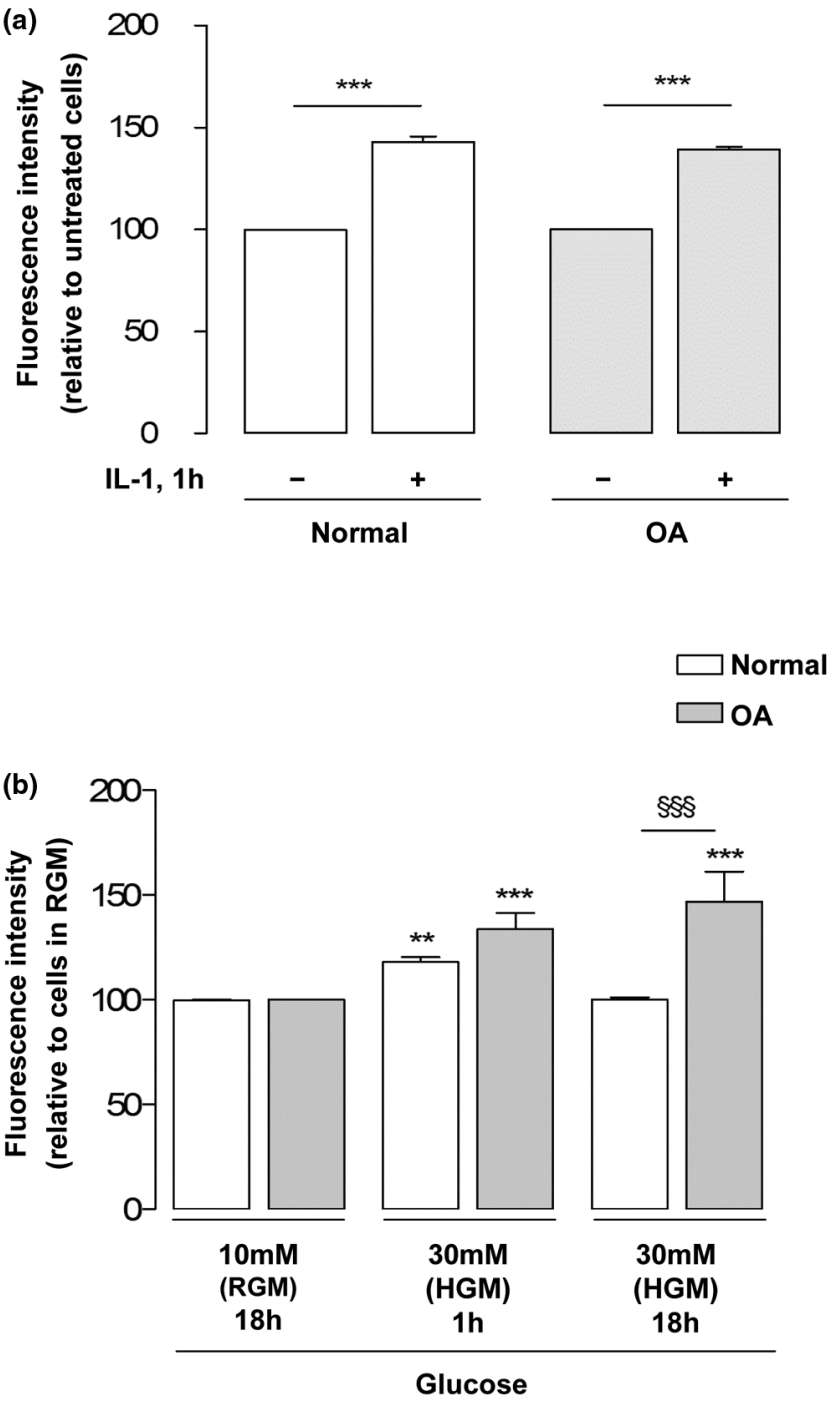
Modulation of reactive oxygen species production by IL-1β and high glucose. **(a) **Reactive oxygen species (ROS) production in normal and osteoarthritis (OA) chondrocytes treated with or without IL-1β 30 ng/ml for 1 hour (n = 4). **(b) **ROS production in normal and OA chondrocytes (n = 5) cultured in regular glucose medium (RGM) or in high glucose medium (HGM, 30 mM) for the periods indicated and then loaded with 5 μM 2',7'-dichlorodihydrofluorescein diacetate for 20 minutes at 37°C, as described in Materials and methods. Results expressed as the percentage relative to the respective control cells maintained in RGM. **P < 0.01 and ***P < 0.001 relative to the respective control cells maintained in RGM, ^§§§^*P *< 0.001 between normal and OA chondrocytes exposed to the same glucose concentration for the same period. Bars = mean ± standard deviation.

As a positive control, normal and OA chondrocyte cultures were treated with IL-1β 30 ng/ml for 1 hour, which increased the fluorescence intensity by approximately 40% relative to the respective control cells (Figure 7a).

Chondrocytes were loaded with the probe to detect ROS production after being cultured under regular or high glucose conditions for 1 hour or 18 hours. The fluorescence intensity detected in each condition was therefore due solely to the amount of ROS produced during the 20-minute incubation with the probe and not to the total amount produced during the previous 1-hour or 18-hour culture periods. In these conditions, the fluorescence intensity of normal and OA chondrocytes that had been incubated in high glucose medium for 1 hour increased similarly when compared with their respective control cells maintained in RGM (Figure 7b). This indicates that exposure of both normal and OA chondrocytes to a high glucose concentration rapidly increases the intracellular production of ROS.

After this initial increase, ROS production returned to control levels in normal chondrocytes that had been cultured under high glucose for 18 hours, while OA chondrocytes still produced increased amounts of ROS, identical to those found in cells that had been cultured under high glucose for only 1 hour (Figure 7b).

## Discussion

The present study has demonstrated that normal and OA chondrocytes isolated from human articular cartilage do not differ in their relative capacity for glucose transport and GLUT-1 content. Moreover, GLUT-1 is constitutively expressed in both normal and OA chondrocytes (Figure 1). The kinetic characteristics of glucose uptake in normal and OA chondrocytes, as reflected by the affinity and maximal velocity values determined, are in the same range, although slightly higher, as those previously reported in bovine chondrocytes [31]. These results are in agreement with other studies [23,32], although GLUT-1 expression in chondrocytes has also been reported to be exclusively inducible [24] and to be either increased [32] or decreased [33] in OA chondrocytes relative to normal cells. The reasons for these discrepancies are unclear, but OA in humans is now understood to be a broad continuum and it is possible that GLUT-1 expression is increased and then decreased at early and later stages of the disease. Alternatively, the observed differences may be related to species investigated or to the culture conditions used in these studies. Nonetheless, we cannot discard the possibility that *in situ *normal and OA human chondrocytes can express distinctly different GLUT-1 levels – especially due to the presence in the OA joint of proinflammatory catabolic cytokines such as IL-1β, which has been shown to induce GLUT-1 expression both in the present study (Figure 2) and in other studies, along with TNF-α and IL-6 [24,25].

Regulation of GLUT-1 has been shown to occur in various cell types and to involve changes at the transcriptional or post-transcriptional levels, depending on the stimulus and cell type considered [27,28,33]. Furthermore, subcellular redistribution between the plasma membrane, intracellular compartments of the Golgi apparatus and protein degradation structures, such as the lysosome, have been shown to mediate high-glucose-induced and low-glucose-induced changes in GLUT-1 protein content and hexose uptake capacity [27,34,35].

In the present study, glucose deprivation similarly upregulated 2-DG transport (Figure 3) and GLUT-1 protein levels (Figure 4) in normal and OA chondrocytes. This upregulation was also observed in other cells, being considered a protective mechanism that maximizes the cell's ability to capture glucose and thus to overcome stressful conditions, such as glucose scarcity or even deprivation [36,37]. Under such conditions, glycogen stores act as a source of sugars [38]. When those stores are depleted, due to persistence or recurrence of glucose shortage or deprivation, a hypoglycosylated form of GLUT-1 accumulates [39] and alternative sources of sugars, such as glycoproteins, may start to be used [38]. In a previous study using the human chondrocytic cell line C-28/I_2_, glucose deprivation elicited the appearance and accumulation of the hypoglycosylated form of GLUT-1 [40]. In the current study, however, no such band was detected in either normal or OA chondrocytes (Figure 4). This indicates that human chondrocytes deprived of glucose can still carry on processes such as protein glycosylation, suggesting they can store more glycogen than transformed C28/I_2 _cells.

In contrast, normal chondrocytes responded to high glucose by decreasing the 2-DG uptake (Figure 3) and the total GLUT-1 content (Figure 4a), suggesting that downregulation of GLUT-1 mediates the decrease in glucose transport. This mechanism can protect articular chondrocytes against the deleterious effects of excessive intracellular glucose accumulation, as seen in other cells [28,41,42]. Accordingly, after the initial increase, ROS production in normal chondrocytes exposed to high glucose concentrations for 18 hours returned to control levels (Figure 7b), accompanying the decrease in GLUT-1 content – whereas ROS production remained elevated in OA chondrocytes (Figure 7b), paralleling their inability to downregulate glucose uptake and the GLUT-1 content (Figures 3 and 4b). Since ROS are involved in the pathophysiology of OA, their prolonged production when OA chondrocytes are exposed to excessive amounts of glucose is likely to directly damage those cells and to aggravate catabolic processes that can contribute to OA progression in diabetic patients. On the other hand, the increased production of ROS observed in normal chondrocytes (Figure 7b), although lasting a shorter time than in OA cells, may not be devoid of deleterious effects, especially if prolonged exposure to high glucose occurs, as may be the case in poorly controlled diabetic patients.

Lysosomal degradation is probably the main mechanism accounting for high-glucose-induced GLUT-1 downregulation in normal chondrocytes, since GLUT-1 mRNA levels remained unchanged (Figure 5a) and only the lysosome inhibitor (chloroquine) effectively counteracted the GLUT-1 decrease (Figure 6). This observation is in agreement with studies in other cells where high glucose, glucose re-feeding after deprivation or diabetic conditions led to GLUT-1 routing to intracellular compartments followed by lysosomal degradation [26,27,34,35]. Since GLUT-1 mRNA levels remained unchanged in OA chondrocytes exposed to high glucose concentrations (Figure 5b), their inability to downregulate the GLUT-1 content (Figure 4b) is probably due to impaired GLUT-1 protein degradation. Further studies are required to identify the primary defect responsible for that impairment, which may lie in any process from glucose sensing and metabolism to GLUT-1 intracellular trafficking and lysosomal degradation. Whether that defect already exists or is induced by exposure to high glucose also warrants further investigation.

From another perspective, the inability of chondrocytes to modulate GLUT-1 gene transcription in response to high glucose concentrations, unlike other cells [28,39], may render chondrocytes especially susceptible to hyperglycemia episodes – especially when the episodes are prolonged, as is often the case in poorly controlled type 2 DM patients. In such circumstances, augmented GLUT-1 degradation may not be sufficient to prevent deleterious increases in the intracellular glucose concentration. This insufficiency is even more striking in OA chondrocytes, which completely failed to downregulate both 2-DG uptake (Figure 3) and GLUT-1 protein (Figure 4b) under high glucose concentrations.

## Conclusions

The present study has shown that normal human chondrocytes adjust to variations in the extracellular glucose concentration by modulating GLUT-1 synthesis and degradation through the lysosome pathway. OA chondrocytes are unable to adjust to high extracellular glucose, however, showing defective GLUT-1 downregulation that leads to the intracellular accumulation of glucose, and increased oxidative stress. This downregulation can constitute an important pathogenic mechanism by which conditions characterized by hyperglycemia, such as DM and other situations involving impaired glucose metabolism, can promote degenerative changes in chondrocytes that facilitate the development and progression of OA.

## Abbreviations

2-DG: 2-deoxy-D-glucose; DM: diabetes mellitus; DMEM: Dulbecco's modified Eagle's medium; GLUT-1: glucose transporter-1; IL: interleukin; NF: nuclear factor; OA: osteoarthritis; PBS: phosphate-buffered saline; PCR: polymerase chain reaction; RGM: regular glucose medium; ROS: reactive oxygen species; RT: reverse transcriptase; TNF: tumor necrosis factor.

## Competing interests

The authors declare that they have no competing interests.

## Authors' contributions

SCR carried out chondrocyte cultures under different glucose concentrations, 2-DG uptake assays, some of the western blots, the ROS production assay and real-time RT-PCR experiments, and participated in the study design and in drafting the manuscript. JG isolated and set up the chondrocyte cultures and performed some experiments. FJ collected normal and OA cartilage and participated in the study design. AM collaborated in the 2-DG uptake assays and the study design, and revised the manuscript. CL participated in the study design. AFM conceived of, designed and coordinated the study, set up some chondrocyte cultures and drafted the manuscript. All authors made intellectual contributions to the project and read and approved the final manuscript.
